# The bedside as a difficult learning environment: overcoming the reluctance to see patients

**DOI:** 10.15694/mep.2017.000020

**Published:** 2017-02-01

**Authors:** Seema Biswas, Itamar Tzadok

**Affiliations:** 1 Ziv Medical Center

**Keywords:** bedside learning, clinical teaching, health disparities, access to healthcare

## Abstract

This article was migrated. The article was marked as recommended.

## Letter

I am increasingly alarmed at the number of medical students who are intimidated at the thought of speaking to patients. Worse still, so many students don't seem to think that taking a history or performing a physical examination is worthwhile. We create educational programmes that emphasise and examine reliable clinical skills in order for students to really understand clinical symptoms and signs and perfect their diagnostic skills. There is more still to gain from a physical closeness to patients, though. Once students are put together with patients, no matter how reluctant the initial introduction, a chemical reaction seems to ensue and a genuine concern and closeness grows. Students learn medicine, yes; but they also embark on their relationships with patients which will one day become the doctor-patient relationship that so much is written about.

We live in an era where more is possible in medicine and health promotion than ever before. It is easy for doctors and students to lose ourselves in the technical capabilities of special investigations and excellence in intervention but the intention was never to lose our bearings with our patients.

The effect of this closeness is to know more about our patients' lives, the impact of illness of our patients and the real causes of their ill health. Students have time to spend at the bedside and may safely visit patients at home. As students care more for their patients, they are more prepared to look after them and see them through some of the worst moments of their lives.

A medical student lists what he has gained from seeing patients in difficult circumstances:

1. Dealing with real patients: I have interviewed several patients only to learn that in spite of what they tell me, they are actually taking several medications, they do, indeed, suffer from oesophageal reflux, and, they have undergone previous surgery, even though, while we speak, they answer 'no' to all my questions. I learned to ask the same question in many different ways. Moreover, I learned that in order to really know the medical history of the patient, I needed to invest time in building a relationship and this relationship has to be "fast-tracked".
2. Making the most the closeness to a patient and influencing the course of a disease: a patient with poorly controlled non-insulin dependent diabetes waited until his wife left the room for a few minutes to ask me a question: "is my inability to have an erection connected to my diabetes? I have not had sex for two years". The patient also suffered from depression and was coping with a sick child at home. I was glad that he asked me; further, I was relieved that he was not about to leave the hospital without mentioning to someone what was really bothering him. He explained that he was asking me this question because he thought this was entirely unrelated to why he would see his doctor. As a student I seemed to be just qualified enough but not too intimidating for the patient to confide in comfortably.
3. Working with the patient's family in order to help the patient: I've learned that the whole family suffers when a patient is unwell. Consulting visiting family to supplement a medical history adds valuable information, but, more than, this adds valuable context. For example, without the assistance of a patient's daughter I would not have known that the patient had collapsed because of days of melaena at home. I would also not have known that, since the death of his wife two years previously, he has been depressed, was drinking heavily (undoubtedly the cause of his bleeding duodenal ulcer) and that, although his family was concerned, they were struggling to help him.
4. Situational awareness and compassion: while I was half-way through a medical interview, the nursing staff came to help the patient take a bath. The patient had undressed, been to the toilet and was about to take a bath, when the medical round arrived at the patient's bed. The nurses said that the patient was about to take a bath but the round continued at the bedside with doctors questioning the students on basic physiology. I tried to hurry the questions along, reassure the patient and move the ward round on so that the patient could continue his bath. I felt the patient's discomfort and found it hard to hide my indignation.
5. Dealing with the realities of providing health services: doing the bare minimum leaves patients lost in hospital systems. At the time a young man with head trauma was being discharged, he was told to consult the Head and Neck specialist. He had the wrong medical documents with him, had no idea where the department was, how to make an appointment, whom to see, and what to tell them. I checked the documents, found the correct documents, accompanied him to the clinic and helped him book an appointment at the correct desk. Ultimately, he was seen on the same day. It occurred to me that this happens to patients all the time.

Doctors have a fundamental role in advocacy and it is never too early for students to learn this. Students study medicine in order to make the world a better place for people to live. We as teachers should be teaching them exactly how to engage with patients and tackle global health problems so that when students become doctors, making the world a better place is exactly what they can do.

No one is closer to patients than their doctors. Patients make up the community and, therefore, no one knows more about the community than doctors. As we observe the politics and current affairs of the world around us, perhaps doctors have a greater role to play in societies that address disparities in the determinants of health as well as access to healthcare. Medical students need to learn to get close to patients.

## Notes On Contributors

Seema Biswas is head of surgical teaching in Ziv Medical Center. Her interests include global health, she is editor-in-chief of BMJ Case Reports and she works as a field surgeon for the International Committee of the Red Cross.

Itamar Tzadok is a medical student in his first medical rotation. He is on a four-year graduate programme at Bar Ilan University. He has a BSc in Chemistry.

## Bibliography/References

## Declarations

The author has declared that there are no conflicts of interest.

