# Supplementary material for: The bedside as a difficult learning environment: overcoming the reluctance to see patients
Source: MedEdPublish (2016). 2017 Feb 1;6:20. [Version 1] doi: 10.15694/mep.2017.000020 (PMC10885222; doi:10.15694/mep.2017.000020)
Supplement: Peer review history

# Reviewer report: Reviewer response for version 1

**DOI:** 10.21956/mep.19418.r28520

**Published:** 2017-02-06

**Authors:** Michelle McLean

**Affiliations:** 1 Bond University

This review has been migrated. The reviewer awarded 4 stars out of 5 Thank you, Seema and Itamar, for providing a student's perspective on what it is like to be at the coal-face of medicine. Students have an important role to play as patient advocates when everyone else appears to be so busy getting through their daily schedule. Compassion and empathy are clearly important. I would recommend that students read this letter.

Reviewer Expertise:

NA

No decision status is available

# Reviewer report: Reviewer response for version 1

**DOI:** 10.21956/mep.19418.r28519

**Published:** 2017-02-04

**Authors:** Trudie Roberts

**Affiliations:** 1 Leeds Institute of Medical Education

This review has been migrated. The reviewer awarded 2 stars out of 5 An interesting contribution. However knowing what training students receive prior to seeing patients would have been useful. Working with patients as part of the teaching Faculty is becoming more common in the UK. this means that they form a part of preparing students to be for the clinical environment. In Leeds students are ultra keen to see and talk to patients from the get go. If I look back at my own experience it was more like that described by the authors but then scant attention was paid to helping students develop communication skills

Reviewer Expertise:

NA

No decision status is available

# Reviewer report: Reviewer response for version 1

**DOI:** 10.21956/mep.19418.r28522

**Published:** 2017-02-02

**Authors:** Richard Hays

**Affiliations:** 1 James Cook University

This review has been migrated. The reviewer awarded 2 stars out of 5 While this letter raises some legitimate issues about potential dilution of clinical experience, my experience has been different. My observation is that once initial under-confidence is overcome and the transition to the clinical student role is over, students are happy to, and enjoy, engaging with patients in both ambulatory and in-patient settings. Other factors may slow the transition down. In-patients are becoming sicker and less able to interact with students and the increasing complexity of many patients can be hard to grasp early on. There also many strong (and important) messages about not harming patients, potentially reducing students to observer roles rather than more active engagement. We clinical teachers need to role model the right behaviours, provide feedback and nudge learners through the transition to clinical roles. This may not be easy to do in busy workplaces where there are many pressures on workloads and deadlines and (inevitably) some institutional politics, but it is our role to help learners understand how to navigate the systems in which we work.

Reviewer Expertise:

NA

No decision status is available

# Reviewer report: Reviewer response for version 1

**DOI:** 10.21956/mep.19418.r28523

**Published:** 2017-02-02

**Authors:** Gert Van Zyl

**Affiliations:** 1 University of the Free State

This review has been migrated. The reviewer awarded 3 stars out of 5 I support the positive comments of the previous reviewers. A very interesting topic and the letter touches on some of the aspects of communication. I wondered what the generational differences have in the outcomes of communication skills or lack there of. It again highlights the importance of communication skills in the teaching and training of health professionals. A topic worth exploring more.

Reviewer Expertise:

NA

No decision status is available

# Reviewer report: Reviewer response for version 1

**DOI:** 10.21956/mep.19418.r28521

**Published:** 2017-02-01

**Authors:** Trevor Gibbs

**Affiliations:** 1 AMEE

This review has been migrated. The reviewer awarded 3 stars out of 5 An interesting letter that highlights very clearly to need to fully appreciate what we mean by communication skills. The comments, presumably from one of the authors who is a medical student explore the holistic nature of communication and how perhaps what we believe has been taught in our communication skills curricula elements are either being taught incorrectly or not fully appreciated by our students. It would be interesting to also read of what sort/what form of communication skills this student had been exposed to, what teaching and learning methodologies had been used and perhaps how they perhaps could have been improved.

Reviewer Expertise:

NA

No decision status is available
